# Co-expression of CXCL8 and HIF-1α is associated with metastasis and poor prognosis in hepatocellular carcinoma

**DOI:** 10.18632/oncotarget.4412

**Published:** 2015-06-10

**Authors:** Xian-Peng Li, Xiao-Yu Yang, Ewelina Biskup, Jiang Zhou, Hong-Liang Li, Yi-Feng Wu, Ming-Liang Chen, Feng Xu

**Affiliations:** ^1^ Division of Gastroenterology and Hepatology, Yinzhou Hospital Affiliated to Medical School of Ningbo University, Ningbo, China; ^2^ Division of Special Treatment II, Eastern Hepatobiliary Surgery Hospital, Second Military Medical University, Shanghai, China; ^3^ Department of Oncology, University Hospital of Basel, Basel, Switzerland; ^4^ Division of Hepatobiliary Surgery, Yinzhou Hospital Affiliated to Medical School of Ningbo University, Ningbo, China

**Keywords:** IL-8, cytokines, migration, hypoxia, metastasis, HCC

## Abstract

Hypoxia inducible factor-1α (HIF-1α), induces cytokines such as CXCL8 and tumor dissemination, chemo- and radio-resistance. We analyzed correlation between HIF-1α and CXCL8 levels, tumor characteristics and overall survival in 102 hepatocellular carcinoma (HCC) patients. Levels of HIF-1α and CXCL8 were increased in HCC tissues and cell lines. Patients with high levels of HIF-1α and CXCL8 had worse outcome and poorer prognosis than those with lower levels. Co-overexpression of HIF-1α and CXCL8 was an independent negative prognostic factor for overall and disease-free survival. HIF-1α silencing and CXCL8 siRNA decreased migration under hypoxic conditions in vitro. Hypoxia-induced activation of AKT/mTOR/STAT3 pathways was reversed by depletion of CXCL8. We conclude that HIF-1α and CXCL8 induce HCC progression and metastasis, associated with activation of AKT/mTOR/STAT3. Co-expression of HIF-1α and CXCL8 is a prognostic marker and a potential therapeutic target in HCC.

## INTRODUCTION

Hepatocellular carcinoma (HCC) is the fifth most common cancer and the third leading cause of cancer mortality worldwide [1-4]. Inflammatory tumor microenvironment is crucial in tumor progression [5-8]. Interleukin-8 (IL-8), also known as CXCL8, is a chemokine with tumorigenic and angiogenic properties [9]. It is secreted by inflammatory and tumor cells. Several studies demonstrated that cells can produce IL-8/CXCL8 in response to inflammation, hypoxia and hyperoxia [10, 11]. IL-8/CXCL8 is associated with tumor angiogenesis, metastasis and poor prognosis in several cancer types [12]. Hypoxia stimulates cancer development, invasion and metastasis. Hypoxia inducible factor-1α (HIF-1α) stimulates cytokines expression, increases tumor dissemination, cell proliferation, angiogenesis and survival [13-16].

Here we investigated the relations between CXCL8 and HIF-1α and evaluated prognostic and therapeutic values of CXCL8 and HIF-1α in a large group of HCC patients based on a decade of follow-up.

## RESULTS

### Upregulated HIF-1α and CXCL8 expression in HCC

**Figure 1:**
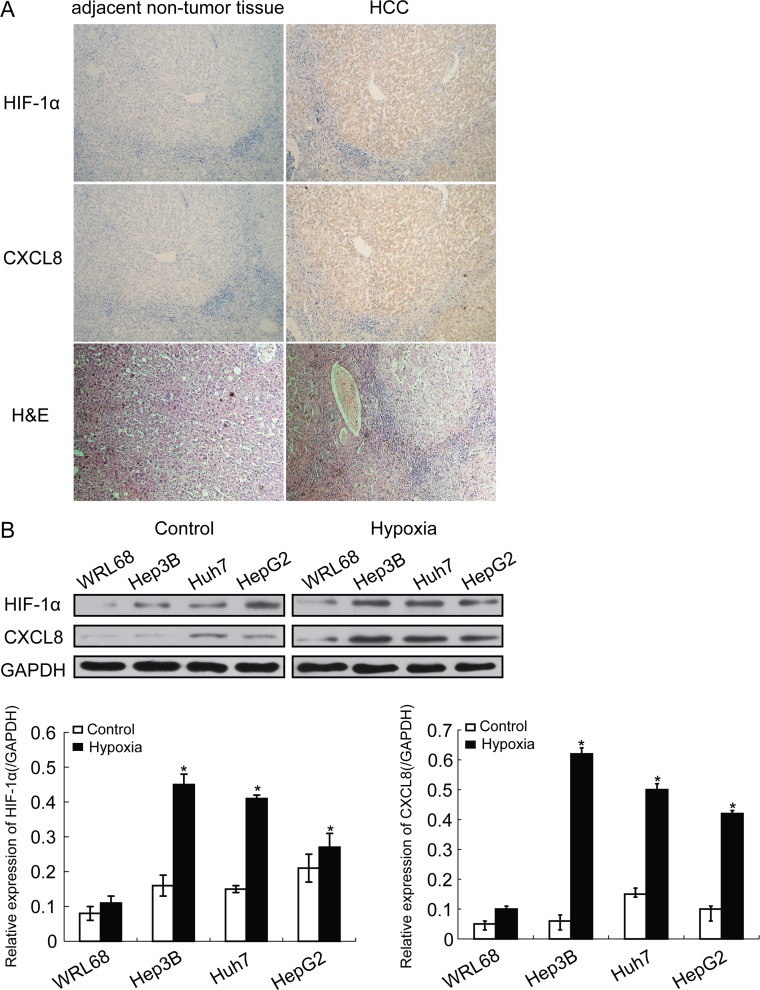
HIF-1α expression is increased in HCC tissues **A.** Representative immunohistochemistry images of human HCC tissues for detection of HIF-1α and CXCL8 (magnification, ×100). **B.** Protein expression of HIF-1α and CXCL8 in HCC cell lines and normal liver cells (*P < 0.05). GAPDH was used as a control.

HIF-1α and CXCL8 were detected in paraffin-embedded serial sections from 102 HCC patients, which underwent hepatectomy. Expression of HIF-1α and CXCL8 was higher in HCC tissues compared to adjacent non-tumor liver tissues (Figure 1A). Similar results were obtained in HCC cell lines including Hep3B, Huh7 and HepG2 compared with normal liver cell line WRL68. For hypoxia conditions, cells were incubated in a humidified Heto multigas incubator in 0.5% O_2_, 5% CO_2_ and 95% N_2_. HIF-1α and CXCL8 was increased in all HCC cell lines but not normal liver cells (Figure 1B).

### HIF-1α promote migration and invasion of HCC cells by regulating the CXCL8 expression

**Figure 2:**
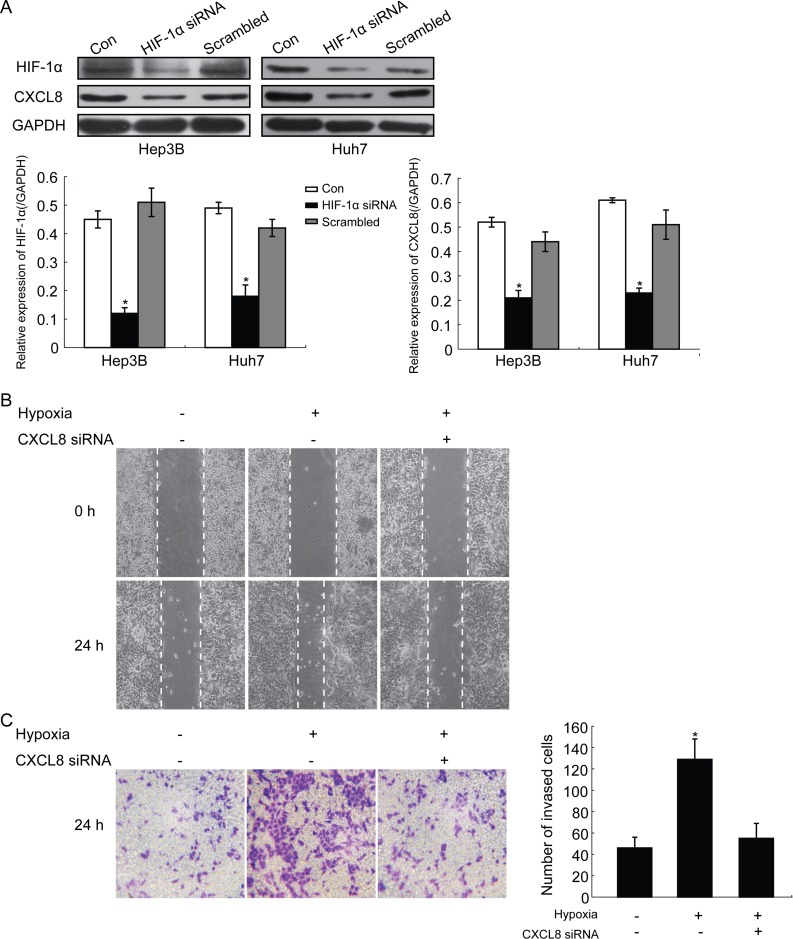
Representative images HIF-1α promotes migration and invasion of HCC cells by regulating CXCL8 expression **A.** Western blot detection of HIF-1α and CXCL8 protein expression in HCC cell lines treated with siRNA against HIF-1α (*P < 0.05). GAPDH was used as a control. **B.** and **C.** wound healing assays and transwell assays show that increased migration and invasion ability of HCC cells under hypoxia conditions was partly reversed by siRNA against CXCL8. The numbers of invasive HCC cells were calculated of ten random microscopic fields. Data shown as the mean±SD of three independent experiments.

Western blot assays were used to evaluate the effect of HIF-1α silencing on the expression of CXCL8 in HCC cells (Hep3B and Huh7). SiRNA silencing of HIF-1α significantly decreased the expression of CXCL8 compared to controls and scrambled groups (Figure 2A). The decrease of HIF-1α was correlated with a down-regulation of CXCL8 expression in HCC cells. Wound healing assays were performed under hypoxia conditions in Hep3B and Huh7. Hypoxic conditions significantly promoted the migration of Hep3B (Figure 2B) and Huh7 (data not shown): Treatment with CXCL8 siRNA inhibited the migration effect (Figure 2B). Transwell assays showed that under hypoxia, cell invasion was increased significantly in Hep3B (Figure 2C) and Huh7 (data not shown) and this effect can be inhibited by CXCL8 siRNA (Figure 2C). These findings indicated that increased expression of HIF-1α under hypoxia via CXCL8 promoted migration and invasion.

### Overexpression of HIF-1α and CXCL8 in HCC is correlated with poor prognosis

**Figure 3:**
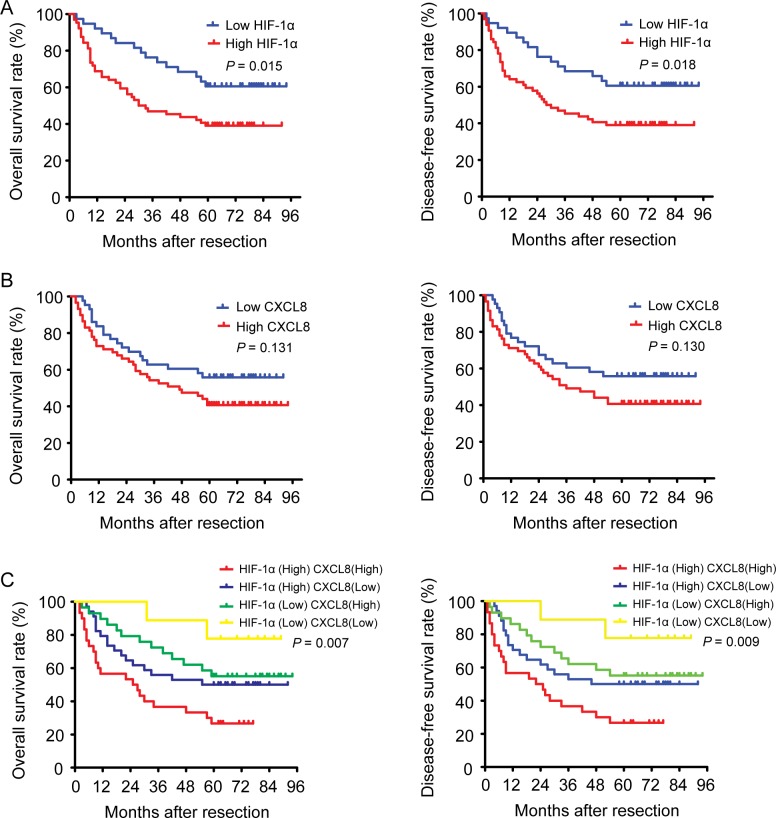
The impact of HIF-1α and CXCL8 expressions on the survival of HCC patients **A.** HIF-1α expression is associated with a poorer overall survival rate and disease-free survival rate. **B.** CXCL8 expression is not related with the overall or the disease-free survival rate. **C.** Both HIF-1α and CXCL8 expression are associated with a poorer overall and disease-free survival rate.

After we first observed a higher HIF-1α and CXCL8 expression in 102 HCC samples (as compared to matched adjacent non-tumor liver tissues (Figure 1A)), we additionally found a correlation between the expression level and tumor features. The expression of HIF-1α was found to be significantly higher in HCC patients with increased tumor size (*P* < 0.001), vascular invasion (*P* = 0.025), intrahepatic (*P* = 0.002) and distant metastasis (*P* < 0.001; Table 1). The expression level of CXCL8 was higher in HCC samples with vascular invasion (*P* = 0.017), intrahepatic (*P* = 0.001) and distant metastasis (*P* < 0.001), and a higher TNM stage (*P* = 0.004; Table 1). Based on these results, we divided 102 HCC patients into 4 groups: high-expression of HIF-1α (*n* = 64), low-expression of HIF-1α (*n* = 38), high-expression of CXCL8 (*n* = 59) and low-expression of CXCL8 (*n* = 43). Patients in the high expression of HIF-1α group had both shorter disease-free survival (DFS, *P* = 0.018) and a worse overall survival (OS, *P* = 0.015) (Figure 3A) than the low-expression group. Interestingly, there is no significant differences between patients with high and low expression of CXCL8 group regarding disease-free survival (*P* = 0.130) or overall survival (*P* = 0.131) (Figure 3B). HCC patients with high-expression of both HIF-1α and CXCL8 had a significantly shorter overall and disease-free survival than patients with sole HIF-1α- or sole CXCL8 high expression (Figure 3C). These observation are suggestive that HIF-1α and CXCL8 expression levels could be valuable predicting factors for recurrence and survival in patients with HCC. Multivariate analysis identified 4 core factors which significantly influenced the overall survival rate and disease-free survival rate (Table 3): double HIF-1α and CXCL8 overexpression, vascular invasion, intrahepatic metastasis and distant metastasis. Co-overexpression of both HIF-1α and CXCL8 was confirmed to be an independent negative factor for overall and diseased-free survival.

**Table 1:** Relationship Between HIF-1α and CXCL8 Expression and Clinicopathologic Features (n = 102)

Variable	HIF-1α Expression			CXCL8 Expression			HIF-1α and CXCL8 Expression		
	Low (n = 38)	High (n = 64)	*P*^a^	Low (n = 43)	High (n = 59)	*P*^a^	Both high (n = 30)	Others (n = 72)	*P*^a^
**Sex**			0.814			0.061			0.803
Male	32	55		40	47		26	61	
Female	6	9		3	12		4	11	
**Age (years)**			0.186			0.352			0.141
≤50	15	34		23	26		11	38	
>50	23	30		20	33		19	34	
**Tumor size (cm)**			**<0.001**			0.779			0.170
≤5	27	7		15	19		7	27	
>5	11	57		28	40		23	45	
**AFP (U/L)**			0.598			0.516			0.835
≤20	6	2		3	5		2	6	
>20	32	62		40	54		28	66	
≤1000	21	35		20	36		18	38	
>1000	17	29		23	23		12	34	
**HBsAg**			0.801			0.399			0.305
Positive	35	58		38	55		26	67	
Negative	3	6		5	4		4	5	
**Anti-HCV**			0.888			0.756			0.261
Positive	1	2		1	2		0	3	
Negative	37	62		42	57		30	69	
**Liver cirrhosis**			0.113			0.326			0.170
Yes	29	39		31	37		17	51	
No	9	25		12	22		13	21	
**Vascular invasion**			**0.025**			**0.017**			**0.002**
Yes	21	49		24	46		27	43	
No	17	15		19	13		3	29	
**Intrahepatic metastasis**			**0.002**			**0.001**			**<0.001**
Yes	5	27		6	26		23	9	
No	33	37		37	33		7	63	
**Distant metastasis**			**<0.001**			**<0.001**			**<0.001**
Yes	2	33		4	31		23	12	
No	36	31		39	28		7	60	
**TNM stage**			0.354			**0.004**			0.325
I-II	31	47		39	39		21	57	
III-IV	7	17		4	20		9	15	
**Edmondson**			0.427			0.109			0.860
I-II	11	14		14	11		7	18	
III-IV	27	50		29	48		23	54	

a Chi-square or Fisher's exact test. AFP, alpha-fetoprotein; HCV, hepatitis C virus; TNM, tumor-node-metastasis.

Statisticaly significant value are given in bold.

### The AKT/mTOR/STAT3 pathway stimulates CXCL8 in hypoxia condition

**Figure 4:**
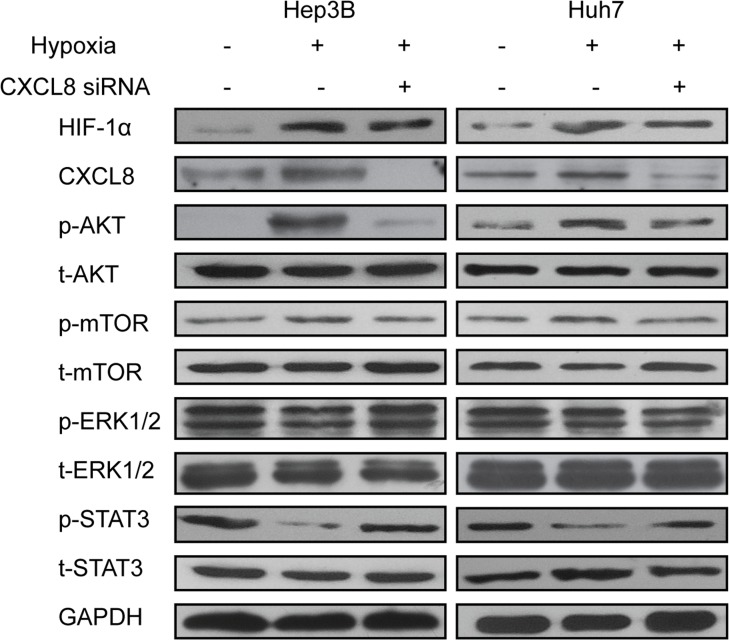
AKT/mTOR/STAT3 signaling pathway was implicated in HIF-1α and CXCL8 synergistic effect Hep3B and Huh7 were transfected with siRNA targeting CXCL8 with or without hypoxia. AKT, ERK, STAT3, mTOR were detected by Western blot.GAPDH was used as control. Results were representated after three independent experiments.

Signaling pathways activated by hypoxia condition were western blot analyzed for expression of phosphorylated AKT, extracellular signal-regulated kinase (ERK), mammalian target of rapamycin (mTOR), signal transducer and activator of transcription 3 (STAT3). In Hep3B and Huh7 cells p-AKT and p-mTOR signals were significantly increased, while p-STAT3 visibly decreased. We did not observe any change in p-ERK expression (Figure 4). Consistently, CXCL8 siRNA was able to markedly reverse the changes of pAKT, ERK and mTOR expression, thus reversing the signaling pathways, which increase the invasion and metastasis features of the HCC cells (Figure 4). These results suggest a mechanism in which hypoxia increases expression of HIF-1α, which then activates AKT/mTOR/STAT3 depending expression of CXCL8.

## DISCUSSION

Hypoxia promotes metastasis and tumor angiogenesis [19-23], in part due to induction of hypoxia inducible factor-1 (HIF-1). HIF-1α regulates hundreds of genes [24 25], increases chemoresistance [26] and radioresistance [28-30], including CXCL8 also known as interleukin-8 (IL-8). CXCL8 is produced by macrophages, endothelial cells, monocytes, neutrophils and fibroblasts. It is proinflammatory [31] and increases chemotaxis, phagocytosis, promoting angiogenesis and inducing processes. Ren Y *et al*. reported a correlation between serum CXCL8 levels and tumor size and tumor stage of HCC [30]. Akiba J *et al*. showed that CXCL8 produced by and suggested that it stimulates HCC cells invasion and metastasis [32]. Cheng XS *et al*. showed that CXCL8 is involved in colorectal cancer [33].

In a large group of HCC patients, we confirmed that HIF-1α and CXCL8 expression is increased in HCC tissues, compared with adjacent non-tumor liver tissues (Figure 1). Expression of HIF-1α was significantly higher in HCC samples with increased tumor size (*p* < 0.001), vascular invasion (*p* = 0.025), intrahepatic (*p* = 0.002) and distant metastasis (*p* < 0.001; Table 1). Levels of CXCL8 were higher in HCC samples with vascular invasion (*P* = 0.017), intrahepatic (*P* = 0.001) and distant metastasis (*P* < 0.001), and a higher TNM stage (*P* = 0.004; Table 1). The Kaplan-Meier analysis showed that HCC patients with high HIF-1α expression or both high expression of HIF-1α and CXCL8 had a worse outcome and prognosis than those with a lower expression (Figure 3). Furthermore, multivariate Cox analysis showed that high expression of both HIF-1α and CXCL8 was an independent prognostic factor of adjusted variables including vascular invasion, intrahepatic metastasis, and distant metastasis in HCC (Table 2). We suggest that expression of HIF-1α and serum levels of CXCL8 are predictive markers of tumor recurrence and prognosis. This biomarkers may help to select patients for more aggressive therapies and to improve the accuracy of the follow-up.

**Table 2:** Multivariate Cox proportional hazards analysis for the candidate variables

Prognostic factors	Overall survival			Disease-free survival		
	HR	95%CI	*P* value	HR	95%CI	*P* value
**Double HIF-1α and CXCL8 (high vs. others)**	2.697	1.590-5.162	0.001	1.997	1.244-4.850	0.002
**Vascular invasion (yes vs. no)**	1.451	1.012-2.457	0.017	1.640	1.038-2.352	0.013
**Intrahepatic metastasis (yes vs. no)**	7.709	4.457-15.244	<0.001	8.511	5.897-15.191	<0.001
**Distant metastasis (yes vs. no)**	2.546	1.428-4.037	0.001	1.809	1.194-4.028	0.006

We further investigated the relationship between HIF-1α and CXCL8 expression and behavior of HCC cells. Silencing of CXCL8 by siRNA under hypoxia decreased cell migration (Figure 2). Inhibition of CXCL8 reversed this effect. AKT promotes tumor metastasis [34]. mTOR, a target of AKT, promotes proliferation, differentiation and inhibits apoptosis [35]. We observed that under hypoxia conditions, HCC cells activate AKT/mTOR/STAT3 pathways. Depletion of CXCL8 reversed these effects (Figure 4).

In conclusion, we showed that increased expression of HIF-1α and CXCL8 in HCC correlate with tumor progression, metastasis and, a poor survival. HIF-1α and CXCL8 are negative prognostic factors, biological markers of tumor invasiveness and therapeutic targets.

## MATERIALS AND METHODS

### Patients and tissue specimens

From April 2002 till March 2014, we conducted a prospective unicentric study. Samples fort he laboratory investigations were collected from April 2002 until December 2008. Formalin-fixed paraffin-embedded tumor tissues and matched adjacent non-tumorous hepatic tissues were collected from 102 HCC patients who underwent hepatectomy as an initial treatment at our hospital. For each patient, the diagnosis of HCC was confirmed on the basis of postoperative pathology (see Figure 1 - representative pathohistologic picture after H&E staining). Preoperatively, no neoadjuvant radio- or chemotherapy was applied, and no invasive interventions, such as percutaneous ablation or chemo-embolization were performed. Each patient was followed up until March 2014. The Yinzhou Hospital Research Ethics Committee approved the research protocol. Written informed consents and voluntary participation in the study were obtained from every patient before the surgery. The clinical baseline characteristics of the HCC patients are presented in Table 1.

### Cell cultures

The human HCC cell lines Hep3B, Huh7, HepG2, and normal liver cell line WRL68 (obtained from Shanghai Institute of Biological Sciences, Chinese Academy of Sciences) were cultured in Dulbecco's modified Eagle's medium, supplemented with 10% FBS (Gibco, USA), in humidified 5% CO_2_, 95% air at 37°C. For hypoxic experiments, cells were incubated in a humidified Heto multigas incubator in 0.5% O_2_, 5% CO_2_ and 95% N_2_.

### Immunohistochemistry (IHC)

The paraffin-embedded tissue specimens were cut into 4-μm serial sections and placed on polylysine-coated slides. After deparaffinization in xylene, sections were rehydrated using a series of graded alcohols and microwave antigen retrievals. Slides were incubated in monoclonal antibodies against HIF-1α (sc-13515, Santa Cruz, USA, 1:200 dilutions) and CXCL8 (ab7747, Abcam, UK, 1:500 dilutions) at 4°C overnight, followed by incubation in the corresponding secondary antibodies at 37°C for 30 min. Staining was performed with DAB and counter-staining with Mayer's hematoxylin. Negative controls were performed by omitting the primary antibodies.

To evaluate the expression of HIF-1α and CXCL8, tissue sections were examined under microscope at a magnification of 200×. Ten fields were randomly selected to count tumor cells and to calculate the percentage of tumor cells with a stronger HIF-1α and CXCL8 expression. In order to quantify the gene expression level, we created a score based on two critera: I. We classified the intensity of HIF-1α and CXCL8 staining according to the following scale: negative = 0; weak = 1; and strong = 2. II. The percentage of immunoreactive tumor cells was calculated and classified on a 5-point scale (0 = 0 %, 1 = 1–25 %, 2 = 26–50 %, 3 = 51–75 %, 4 = 76–100 %) [17, 18]. For statistical analysis, a final score of 0–1 indicates low gene expression; a score of 2–4 indicates high expression of HIF-1α and CXCL8.

### Western blot assay

Cell lysates were subjected to 10 % PAGE and transferred to nitrocellulose filter membranes. The membranes were blocked for 1 h in 5 % non-fat dry milk diluted with TBST (10 mM Tris–HCl and 0.05 % Tween 20). The membranes were then incubated with primary antibodies at 4 °C overnight, followed by incubation with appropriate secondary antibodies at room temperature for 2 h. The primary antibodies were mouse monoclonal anti-HIF-1α (sc-13515, Santa Cruz, USA, 1:1000 dilutions), mouse monoclonal anti-CXCL8 (ab7747, Abcam, UK, 1:1000 dilutions), rabbit polyclonal anti-t-AKT (sc-8312, Santa Cruz, USA, 1:1000 dilutions), rabbit polyclonal anti-p-AKT (sc-135650, Santa Cruz, USA, 1:1000 dilutions), rabbit polyclonal anti-t-ERK1/2 (sc-292838, Santa Cruz, USA, 1:1000 dilutions), rabbit polyclonal anti-p-ERK1/2 (sc-101760, Santa Cruz, USA, 1:1000 dilutions), rabbit polyclonal anti-t-mTOR (sc-8319, Santa Cruz, USA, 1:1000 dilutions), rabbit polyclonal anti-p-mTOR (sc-101738, Santa Cruz, USA, 1:1000 dilutions), rabbit polyclonal anti-t-STAT3 (sc-482, Santa Cruz, USA, 1:1000 dilutions), rabbit polyclonal anti-p-AKT (sc-135649, Santa Cruz, USA, 1:1000 dilutions) and mouse monoclonal anti-GAPDH (sc-365062, Santa Cruz, USA, 1:5000 dilutions). The membranes were washed three times with PBS, and the immunoreactive bands were visualized using an ECL plus Kit, according to the manufacturer's instructions. GAPDH was used as a gel loading control.

### Small interfering RNA (siRNA) and transient transfection

siRNA targeting HIF-1α was generated with the sequences 5′-GUGAUGAAAGAAUUACGAAUTT-3′ and 5′-AUUCGGUAAUUCUUUCAUCACTT-3′, which were used to generate pSilencer3.1-HIF-1α. A non-functional siRNA (scrambled sequence: 5′-UUCUCCGAACGUGUCACGUdTdT-3′; 5′-ACGUGACACGUUCGGAGAAdTdT-3′) was also produced. CXCL8 siRNA was purchased from Santa Cruz (sc-39631, USA). The siRNA transfection was optimized using Lipofectamine 3000 (Invitrogen, Carlsbad, CA, USA), according to the manufacturer's instructions. 24-48 h after transfection, cells were analyzed using the assays described below.

### Wound healing assays and transwell assays

For wound healing assays, cells were seeded in six-well plates to a confluency of approximately 60-70%. Wounds were created in the cell monolayer with a 200-μl pipette tip and the indicated plasmids were transfected into cells. Dead cells were eliminated with PBS wash. Wound closure was monitored at 0 and 24 h. Cell invasion assays were evaluated using transwell chamber assay (Millipore, Billerica, MA, USA) according to the manufacturer's instruction. Matrigel (BD Biosciences, USA) was left to polymerize at the base of the top chamber of a 24 well Transwell plate (8 μm, Corning Costar Corp, USA) for 45 min at 37°C. Cells (5 × 10^4^ cells/well) were exposed to starvation by eliminating serum and growth factor for 24 h and then added to the top chambers. The bottom chambers were filled with serum-containing medium. Cultures were maintained for 48 h. Cells adherent to the upper surface of the filter were removed using a cotton applicator, and then stained with crystal violet. Cells were counted in 10 random fields at ×100 magification and the mean±SD was calculated. To assure a representative conduct of the assays, they were performed in triplicate wells and repeated twice.

### Statistical analysis

Statistical analyses were performed using SPSS 18.0. Chi-square tests and Fisher's exact tests to compare the clinicopathological data. Kaplan-Meier analysis was used to estimate survival rates and the two-sided log-rank test was used to compare differences. Univariate and multivariate analyses were based on a Cox proportional hazard regression model. *In vitro* data were analyzed using one-way ANOVA method. A *P* value <0.05 was considered statistically significant.
